# The feasibility of [^18^F]EF5-PET/CT to image hypoxia in ovarian tumors: a clinical study

**DOI:** 10.1186/s13550-020-00689-z

**Published:** 2020-09-10

**Authors:** Maren Laasik, Johanna Hynninen, Sarita Forsback, Tommi Noponen, Marko Seppänen, Sakari Hietanen

**Affiliations:** 1 Department of Obstetrics and Gynecology, Turku University Hospital, Turku, Finland; 2 Department of Chemistry, Turku PET Centre, University of Turku, Turku, Finland; 3 Department of Nuclear Medicine, Turku PET Centre, University of Turku, Turku, Finland

**Keywords:** EF5-PET/CT, Ovarian cancer, Hypoxia

## Abstract

**Rationale:**

Evaluation of the feasibility of [^18^F]EF5-PET/CT scan in identifying hypoxic lesions in ovarian tumors in prospective clinical setting.

**Methods:**

Fifteen patients with a suspected malignant ovarian tumor were scanned with [^18^F]EF5 and [^18^F]FDG-PET/CT preoperatively. The distribution of [^18^F]EF5-uptake, total intraabdominal metabolic tumor volume (TMTV), and hypoxic subvolume (HSV) were assessed.

**Results:**

[^18^F]EF5-PET/CT suggested hypoxia in 47% (7/15) patients. The median HSV was 87 cm^3^ (31% of TMTV). The [^18^F]EF5-uptake was detected in primary tumors and in four patients also in intra-abdominal metastases. The [^18^F]EF5-uptake in cancer tissue was low compared to physiological excretory pathways, complicating the interpretation of PET/CT images.

**Conclusions:**

[^18^F]EF5-PET/CT is not feasible in ovarian cancer imaging in clinical setting due to physiological intra-abdominal [^18^F]EF5-accumulation. However, it may be useful when used complementarily to FDG-PET/CT.

## Introduction

Ovarian cancer (OC) is the most lethal gynecological malignancy, and the majority of patients are diagnosed at an advanced stage [1]. Although OC is initially chemosensitive, most women experience multiple and finally chemoresistant relapses. The survival odds have not markedly improved despite extensive research, and completeness of surgery is still a major prognostic factor [2].

The presence of hypoxic regions in solid tumors is associated with a poor prognosis for many cancer types [3–5]. Hypoxia-mediated chemoresistance is also the greatest clinical challenge in OC [6, 7].

There is a considerable need for non-invasive imaging of tumor hypoxia since it provides additional information, which could be integrated into strategies of treatment [8]. The method can improve therapeutic outcomes by predicting chemoresistance and selecting potentially treatment-resistant tumors for targeted surgery.

^18^F-nitroimidazolpentafluoropropylacetamide ([^18^F]EF5) is one of the extensively investigated and clinically tested tracers of tissue hypoxia [9, 10]. ^18^F]EF5 belongs to the nitroimidazole group and has considerable membrane permeability and capability to accumulate in viable hypoxic, though not in apoptotic or necrotic cells [11–13].

Since there is no systematic data evaluating the eligibility of hypoxia-imaging among patients with ovarian malignancy, we conducted the prospective clinical study to evaluate the feasibility of [^18^F]EF5-PET/CT scan in identifying hypoxic lesions in ovarian tumors.

## Material and methods

### Study population

This prospective non-randomized study was conducted at Turku University Hospital, Finland, between November 2017 and June 2019. Patients between 38 and 79 years of age with ovarian tumor, who were not pregnant, nursing, or had a history of previous malignancies were included. Ethical approval *was obtained* from the institutional review board (18.10.2016§443), and all subjects signed an informed consent form, ClinicalTrials.gov identifier: NCT04001023.

A whole-body contrast-enhanced [^18^F]FDG-PET/CT and [^18^F]EF5-PET/CT of the abdomen were performed on separate days preoperatively. PET/CT images were then evaluated by a nuclear medicine specialist and gynecological oncologist to assess the distribution of the cancer and to determine regions of suspected hypoxia in the intraabdominal tumor load for targeted biopsies for future research.

### PET/CT scanning procedure

The PET/CT studies were performed with a digital PET/CT scanner: Discovery MI (General Electric Medical Systems, Milwaukee, WI, USA). It has combined PET/CT-scanners with a 128-slice CT and a 3D PET imaging capability. The PET imaging field of view (FOV) was 70 cm in diameter and 20 cm in axial length. To obtain attenuation correction for 511 keV photon distribution, the transmission scan was performed using a low-dose (noise index 30, automatic 3D current modulation, 10–120 mAs, and 120 kVp) CT protocol.

The patients received an intravenous injection of 370 MBq of ^18^F-EF5. A static emission scan was acquired 180 min from the tracer injection to cover the entire abdomen (3 bed positions, 7.5 min/bed). The patients voided prior to the scan. The sinogram data was corrected for deadtime, decay, and photon attenuation and reconstructed in a 256 × 256 matrix. Image reconstruction followed the Q.Clear method (a Bayesian-penalized likelihood reconstruction algorithm for PET) incorporating random and scatter correction with *β* value of 350. The final in-plane FWHM (full-width half-maximum) of the systems was < 5 mm.

A whole-body [^18^F]FDG-PET/CT with low-dose CT combined with diagnostic contrast-enhanced imaging for anatomical reference was performed following the standard institutional protocol for OC. The details of the protocol used for [18F]FDG-PET/CT are the same as abovementioned 18F]EF-5 PET/CT protocol except the larger scan area starting 60 min post injection from the base of the scull to the middle thigh (6 bed positions, 2 min/bed) with 4 MBq/kg of [18F]FDG.

The scans were performed in a random order depending on the availability of the [^18^F]EF5 and camera. The mean interval between scans was 2 (range 1–7) days.

### Image analyses

Total metabolic tumor volume (MTV), maximal standardized [^18^F]FDG, and [^18^F]EF5-uptake values (SUVmax) were assessed with the PET VCAR (Volume Computer-Assisted Reading) program. For [^18^F]FDG-PET/CT analyses the SUVmax values were corrected for body weight and injected dose. TMTV was defined as the sum volume of all lesions.

A hypoxic voxel was defined using a threshold tumor to gluteus maximus muscle ratio (TMR) for [^18^F]EF5-uptake of 1.5, based on earlier experience [14]. The hypoxic subvolume (HSV) was defined as the sum volume of all lesions with TMR over 1.5.

### Statistical analyses

Statistical analyses were performed using JMP Pro 13 software from SAS. Continuous variables were compared using a Wilcoxon rank-sum test. A non-parametric Spearman rank correlation test was used to evaluate the association between SUVmax values. Two-tailed *P* values < 0.05 were considered statistically significant.

## Results

Fifteen patients were enrolled. EF5-PET/CT suggested hypoxia in 46% (7/15) of the patients. In those patients the mean HSV was 87 [Cl95% (9.5–238)] cm^3^ and proportionally 31 [Cl95% (2–47)]% of MTV.

Patients’ clinical and imaging characteristics are presented in Table 1. The distribution of [^18^F]EF5-avid lesions was variable. In 7 of the 15 (46%) patients, [^18^F]EF5PET/CT was suggestive for hypoxia. All of these patients had [^18^F]EF5-accumulation inside the primary tumor and 4 also had [^18^F]EF5-avid metastatic lesions. Seven patients had omental metastases, and 3 had [^18^F]EF5-accumulation in the omental cake.

**Table 1:** Patient characteristics and PET information

Nr.	Age at diagnosis (years)	Weight (kg)	Histology	Stage	MTV (cm^3^)	Hypoxia in [^18^F]EF5 PET/CT	Hypoxic subvolume (cm^3^, % of MTV)	Injected [^18^F]EF5 activity (Mbq)
1	62	106	high grade serous	IIIC	571	yes	364 (64%)	381
2	56	81	endometrioid	IIB	250	yes	121 (49%)	367
3	65	100	sarcoma	IIB	1908	yes	42 (2%)	374
4	38	93	mucinous	IA	158	no		367
5	73	54	carcinosarcoma	IIIC	267	yes	17 (6%)	301
6	79	68	high grade serous	IVB	615	yes	202 (33%)	220
7	67	63	high grade serous	IIIC	955	no		385
8	56	70	fibroma	-	224	no		377
9	50	103	clear cell	IIIC	116	no		378
10	69	77	high grade serous	IVA	324	no		380
11	45	91	serous BOT	IA	66	no		374
12	65	69	high grade serous	IIIC	273	no		377
13	50	152	endometrioid	IIA	173	no		363
14	68	59	high grade serous	IIIC	1288	yes	87 (7%)	362
15	64	60	endometrioid	IIA	346	yes	33 (10%)	177

*BOT* borderline ovarian tumor, *MTV* metabolic tumor volume, *HSV* hypoxic subvolume

**Fig. 1:**
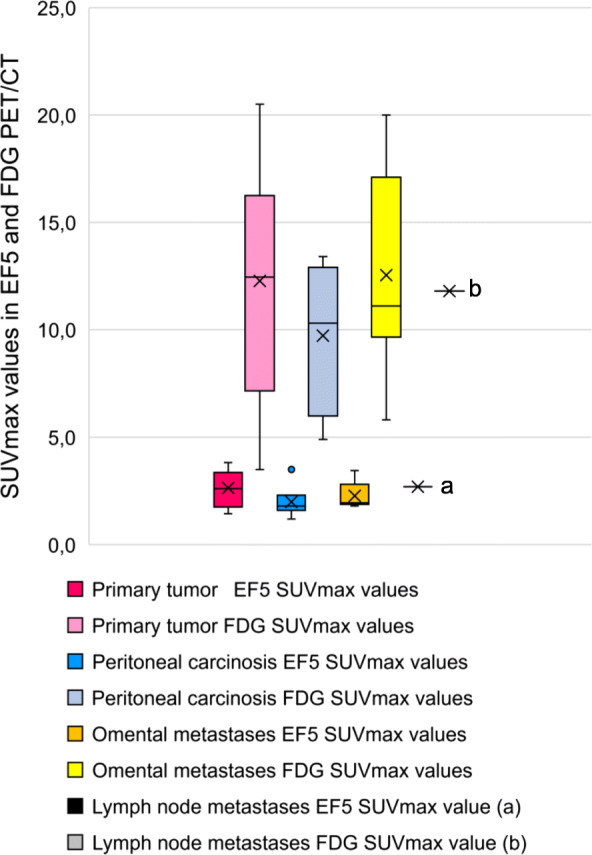
EF5 and FDG SUVmax values in malignant primary tumors and metastases with different anatomical location

All of the [^18^F]EF5-avid tumors also had increased uptake of [^18^F]FDG; however, only a weak statistical correlation between [^18^F]EF5 and [^18^F]FDG-uptake in tumors was detected (Spearman’s rho 0.33, ***p*** = 0.057). SUVmax values of EF5 and FDG uptake in the malignant primary tumors and metastases of 13 patients are presented in Fig. 1.

**Fig. 2:**
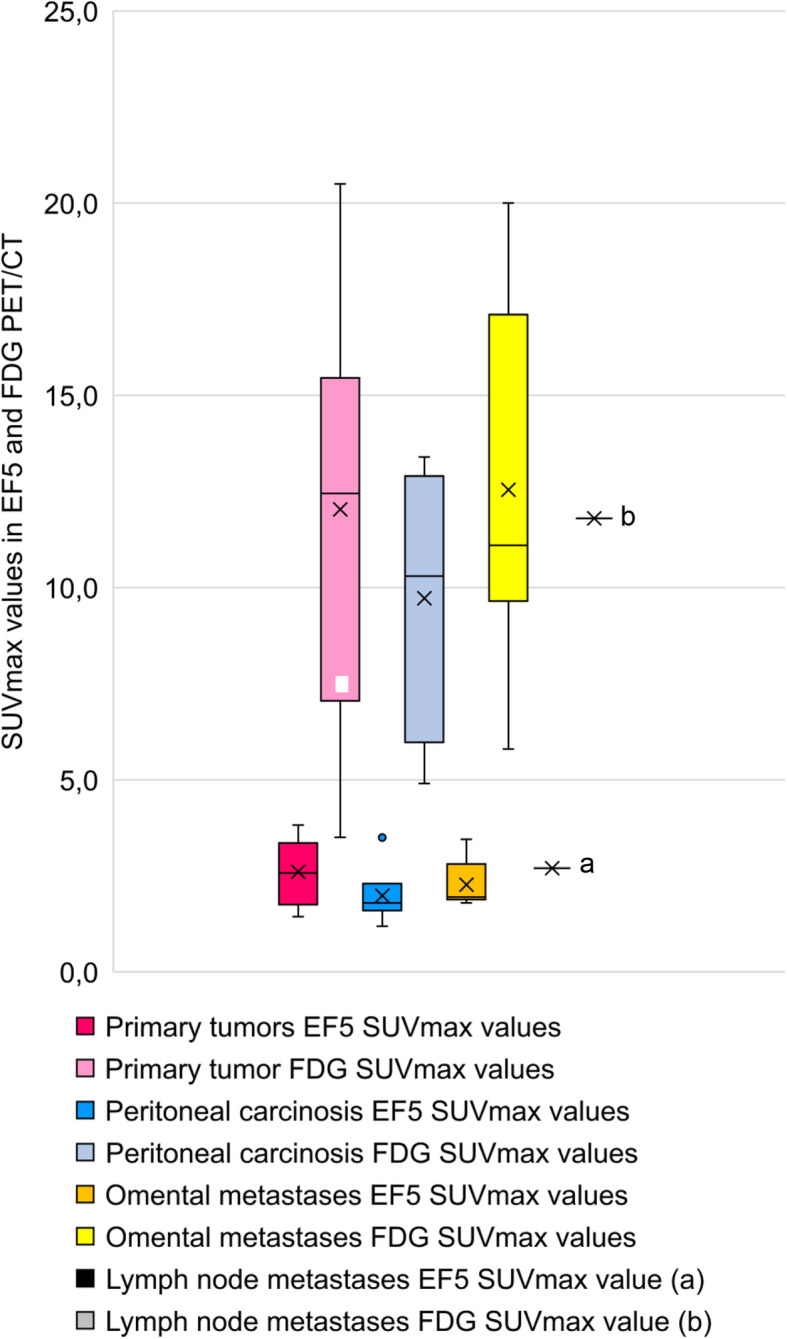
MTV (metabolic tumor volume, cm^3^) and HSV (hypoxic subvolume, cm^3^) of a particular patient (nr 1–15). Under the *x*-axis, the same patient’s distribution of the disease is presented

Seven patients had FDG-avid peritoneal carcinosis, a finding typical of OC. Only 1 patient had [^18^F]EF5-accumulation in the peritoneal carcinosis (thickness < 1 cm). The distribution of the disease, MTV, and HSV values are presented in Fig. 2. None of the patients had [^18^F]EF5-avid metastases when the primary tumor PET-finding was not suggestive of hypoxia. There was no statistical difference between [^18^F]EF5-uptake in the primary tumor and metastases (***p*** = 0.72).

Our study included two patients with non-malign tumors (patient nr 8 and 11), which presented no [^18^F]EF5-accumulation.

**Fig. 3:**
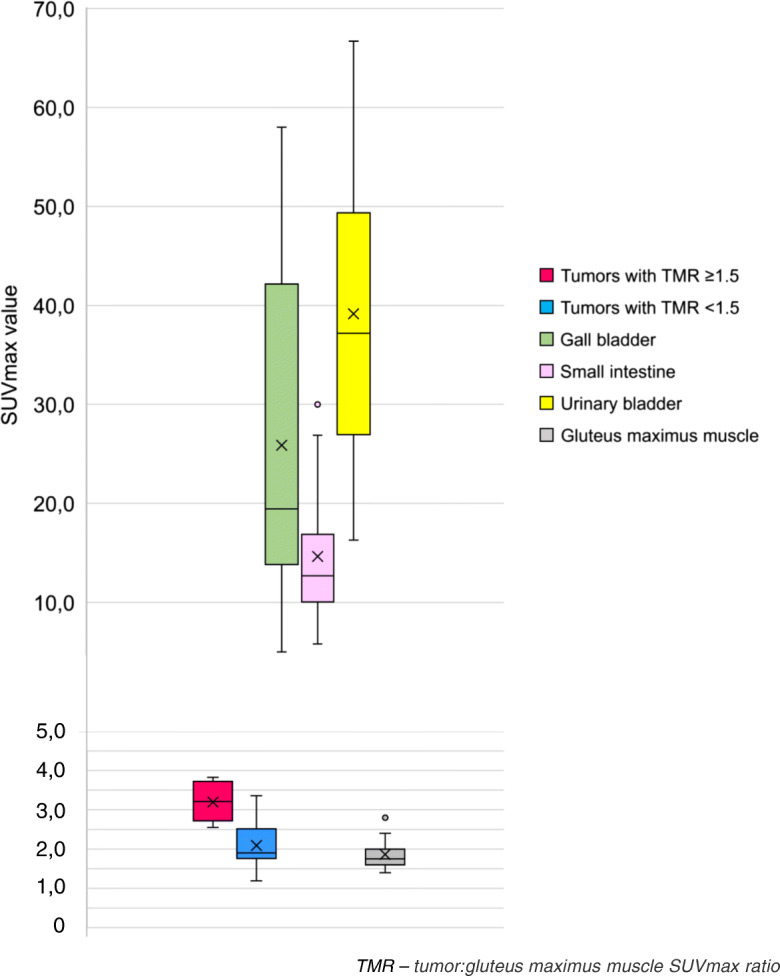
The SUV max values from different anatomical sites in [^18^F]EF5-PET/CT in patients with ovarian tumor

The physiological uptake of [^18^F]EF5 in the gall bladder/bile, small intestine, and urinary bladder was notably higher than in the tumors (Fig. 3).

**Fig. 4:**
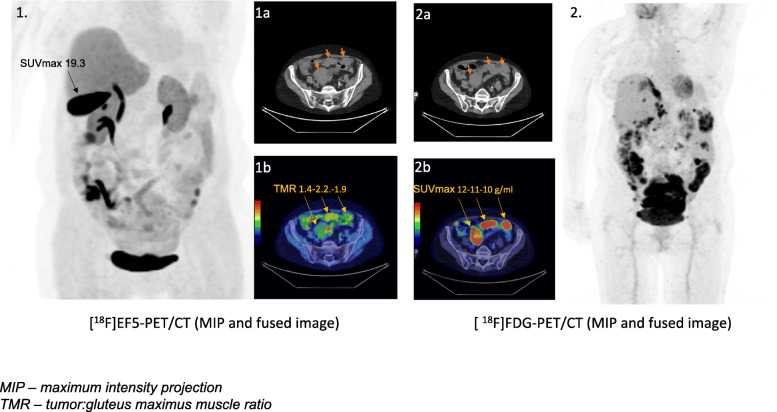
An image of a patient with advanced ovarian cancer. All three metastases are FDG-avid (2b) and 2 of them have also accumulated EF5 (1b). The SUVmax-values in [^18^F]EF5-avid (tumor:gluteus maximus muscle ratio ≥ 1.5) are remarkably lower (1b) compared to the physiological uptake in bile (1.)

A demonstrative EF5- and FDG-PET/CT images of a patient with advanced ovarian cancer are presented in Fig. 4.

## Discussion

Hypoxia is a common phenomenon in cancer with 50–60% of solid tumors containing hypoxic regions [15]. While hypoxia has a well-established role in promoting hematogenous metastases of cancer cells [16], the hematogenous spread is rare in OC at the time of diagnosis [17]. It should also be noted that the role of hypoxia in the transcoelomic spread to the peritoneum and omentum (common to OC) has not been widely investigated. On the basis of our study, [^18^F]EF5-PET/CT suggested hypoxia in half of the patients and the distribution of [^18^F]EF5-uptake was variable. [^18^F]EF5-uptake was detected mainly inside the ovarian tumor and less often in metastases. One preclinical study suggested a hypoxic environment to induce omental/peritoneal metastases [18]*.* Another study [19] which included two OC patients detected EF5-uptake and severe hypoxia in a peritoneal carcinosis biopsied laparoscopically promptly after the injection of EF5. Our cases with widespread peritoneal carcinosis typically had several [^18^F]FDG-avid areas but only one patient had [^18^F]EF5-avid peritoneal lesion.

The previous hypoxia imaging studies are conducted mostly on solid and locally advanced tumors [3, 5, 14, 20, 21], while half of our patients had disseminated cancer in the abdominal cavity hampering the comparison of our results to the other studies. However, [^18^F]EF5-uptake values in our study were comparable to previously reported [^18^F]EF5 SUVmax values in head and neck tumor studies, where the tumor hypoxia was confirmed with immunohistochemical staining [3, 14, 20]. Our study suggests that hypoxia is not a common phenomenon OC in transcoelomic metastases.

According to our study, the feasibility of [^18^F]EF5 PET/CT in ovarian cancer imaging in clinical practice appears to be limited. An uptake of [^18^F]EF5 in tumors was relatively weak and as the tumors were also [^18^F]FDG-avid, the visual estimation of [^18^F]-EF5-PET/CT was strongly lead by [^18^F]FDG-PET/CT. The avidity to both [^18^F]EF5 and [^18^F]FDG can be explained with Warburg’s effect [22], where cancer cells rely widely on glycolysis and reduce their respiration regardless of tissue oxygenation level. Unlike OC, head and neck and lung cancer are isolated tumors that are not surrounded by physiologically EF5-affine tissues. Our study is prospective, and two tumors eventually appeared to be benign. Nevertheless, we consider it important to present them especially as they showed no EF5 uptake.

Previously, two excretory paths of highly lipophilic [^18^F]EF5-tracer have been demonstrated [23, 24]. In the latter, it was assumed that due to slow tracer biliary excretion, only small amounts of activity would be seen in the small intestine. However, our study revealed an excessive [^18^F]EF5-uptake in the bile and small intestine. In contrast to the supradiaphragmatic tumors, this phenomenon imposes limitations on assessing tumors presenting weak [^18^F]EF5-uptake. Especially when located near to the intestine, metastases may be easily be mistaken to physiological uptake and remain unnoted.

## Conclusion

Non-invasive hypoxia imaging with [^18^F]EF5-PET/CT is possible, but its clinical use is restrained by the weak tumor uptake of the tracer compared to the non-specific uptake in excretory organs. The potential usefulness of with [^18^F]EF5-PET/CT in OC could be complementary to FDG-PET/CT with the intent to determine high-risk patients. The role of hypoxia in OC is intensively studied and [^18^F]EF5-PET/CT forms an attractive tool for patient stratification.

## Data Availability

The datasets used and analyzed during the current study are available from the corresponding author on reasonable request.

## Abbreviations

OC: Ovarian cancer
[^18^F]EF5: ^18^F-nitroimidazolpentafluoropropylacetamide
MTV: Total metabolic tumor volume
VCAR: Volume Computer-Assisted Reading
SUVmax: Maximal standardized uptake value
TMR: Tumor to gluteus maximus muscle ratio
HSV: Hypoxic subvolume

## References

[1] Siegel RL, Miller KD, Jemal A (2017). Cancer statistics, 2017. CA Cancer J Clin.

[2] Bristow R, Tomacruz R, Armstrong DK, Trimble E, Montz F (2002). Survival effect of maximal cytoreductive surgery for advanced ovarian carcinoma during the paltinum era: a meta-analysis. J Clin Oncol..

[3] Komar G, Lehtiö K, Seppänen M, Eskola O, Levola H, Lindholm P, et al. Prognostic value of tumour blood flow, [18F]EF5 and [18F]FDG PET/CT imaging in patients with head and neck cancer treated with radiochemotherapy. Eur J Nucl Med Mol Imaging. 2014:2042–50. 10.1007/s00259-014-2818-3 24898846

[4] Kinoshita T, Fujii H, Hayashi Y, Kamiyama I, Ohtsuka T, Asamura H. Lung cancer prognostic significance of hypoxic PET using 18 F-FAZA and 62 Cu-ATSM in non-small-cell lung cancer. 2016;91:56–66. 10.1016/j.lungcan.2015.11.020 26711935

[5] Qian Y, Eyben R Von, Liu Y, Chin FT, Miao Z, Apte S, et al. F-EF5 PET-based imageable hypoxia predicts local recurrence in tumors treated with highly conformal radiation therapy. Radiat Oncol Biol. 2018;1–10. Available from: 10.1016/j.ijrobp.2018.03.045. 10.1016/j.ijrobp.2018.03.045 29859786

[6] Holmes D (2015). Ovarian cancer: beyond resistance. Nature..

[7] Shen W, Li H, Liu L, Cheng J (2017). Expression levels of PTEN, HIF-1 α, and VEGF as prognostic factors in ovarian cancer. Eur Rev Med Pharmacol Sci..

[8] Wilson WR, Hay MP. Hypoxia influences many aspects of the biology of tumours and their responses to therapy. Nat Publ Gr. 2011;11.

[9] Ziemer LS, Evans SM, Kachur A V, Shuman AL, Cardi CA, Jenkins WT, et al. Original article noninvasive imaging of tumor hypoxia in rats using the 2-nitroimidazole 18 F-EF5. 2003;30(2). 10.1007/s00259-002-1037-5 12552344

[10] Gaertner FC, Souvatzoglou M, Brix G, Beer AJ. Imaging of hypoxia using PET and MRI. Curr Pharm Biotechnol. 2012 [cited 2019 Dec 19];13(4):552–570. Available from: http://www.ncbi.nlm.nih.gov/pubmed/22214501. 10.2174/138920112799436267 22214501

[11] Chapman JD, Franko AJ, Sharplin J (1981). A marker for hypoxic cells in tumours with potential clinical applicability. Br J Cancer..

[12] Koch CJ, Scheuermann JS, Divgi C, Judy KD, Kachur AV, Freifelder R (2010). Biodistribution and dosimetry of 18F-EF5 in cancer patients with preliminary comparison of 18F-EF5 uptake versus EF5 binding in human glioblastoma. Eur J Nucl Med Mol Imaging..

[13] Rasey JS, Grunbaum Z, Magee S, Nelson NJ, Olive PL, Durand RE (1987). Characterization of radiolabeled fluoromisonidazole as a probe for hypoxic cells. Radiat Res..

[14] Komar G, Seppänen M, Eskola O, Lindholm P, Grönroos TJ, Forsback S (2008). 18F-EF5: a new PET tracer for imaging hypoxia in head and neck cancer. J Nucl Med..

[15] Vaupel P, Mayer A. Hypoxia in cancer: significance and impact on clinical outcome. Cancer Metastasis Rev. 2007[cited 2019 Sep 16];26(2):225–39. Available from: http://link.springer.com/10.1007/s10555-007-9055-1. 10.1007/s10555-007-9055-1 17440684

[16] Rankin EB, Giaccia AJ. Hypoxic control of metastasis. Science. 2016 [cited 2019 Sep 16];352(6282):175–180. Available from: http://www.ncbi.nlm.nih.gov/pubmed/27124451. 10.1126/science.aaf4405 PMC4898055 27124451

[17] Cormio G, Rossi C, Cazzolla A, Resta L, Loverro G, Greco P, et al. Distant metastases in ovarian carcinoma. Int J Gynecol Cancer. [cited 2019 19];13(2):125–129. Available from: http://www.ncbi.nlm.nih.gov/pubmed/12657111. 10.1046/j.1525-1438.2003.13054.x 12657111

[18] Natarajan S, Foreman KM, Soriano MI, Rossen NS, Shehade H, Fregoso DR, et al. Collagen remodeling in the hypoxic tumor-mesothelial niche promotes ovarian cancer metastasis. Cancer Res [Internet]. 2019 [cited 2019 Sep 16];79(9):2271–84. Available from: http://www.ncbi.nlm.nih.gov/pubmed/30862717. 10.1158/0008-5472.CAN-18-2616 PMC6822898 30862717

[19] Busch TM, Hahn SM, Wileyto EP, Koch CJ, Fraker DL, Zhang P (2004). Hypoxia and photofrin uptake in the intraperitoneal carcinomatosis and sarcomatosis of photodynamic therapy patients. Clin Cancer Res..

[20] Silvoniemi A, Suilamo S, Laitinen T, Forsback S. Repeatability of tumour hypoxia imaging using [ 18 F ] EF5 PET/CT in head and neck cancer. Eur J Nucl Med Mol Imaging [Internet]. 2017; Available from: 10.1007/s00259-017-3857-3%0AORIGINALARTICLE%0ARepeatability. 10.1007/s00259-017-3857-3 PMC5745570 29075831

[21] Yapp DTT, Woo J, Kartono A, Sy J, Oliver T, Skov KA, et al. Non-invasive evaluation of tumour hypoxia in the Shionogi tumour model for prostate cancer with 18 F-EF5 and positron emission tomography. BJU Int. 2007 1 [cited 2019 Sep 19];99(5):1154–60. Available from: http://doi.wiley.com/10.1111/j.1464-410X.2007.06761.x. 10.1111/j.1464-410X.2007.06761.x 17309552

[22] Warburg O (1924). Über den Stoffwechsel der Carcinomzelle. Naturwissenschaften..

[23] Eskola O, Grönroos TJ, Forsback S, Tuomela J, Komar G, Bergman J (2012). Tracer level electrophilic synthesis and pharmacokinetics of the hypoxia tracer [ 18F]EF5. Mol Imaging Biol..

[24] Lin LL, Silvoniemi A, Stubbs JB, Rengan R, Suilamo S, Solin O (2012). Radiation dosimetry and biodistribution of the hypoxia tracer ^18^ F-EF5 in oncologic patients. Cancer Biother Radiopharm.

